# Complicated malaria in children and adults from three settings of the Colombian Pacific Coast: A prospective study

**DOI:** 10.1371/journal.pone.0185435

**Published:** 2017-09-25

**Authors:** Myriam Arévalo-Herrera, Lina Rengifo, Mary Lopez-Perez, Maria I. Arce-Plata, Jhon García, Sócrates Herrera

**Affiliations:** 1 Caucaseco Scientific Research Center, Cali, Colombia; 2 Faculty of Health, Universidad del Valle, Cali, Colombia; Centro de Pesquisas Rene Rachou, BRAZIL

## Abstract

**Background:**

Complicated malaria remains an important public health problem, particularly in endemic settings where access to health services is limited and consequently malaria fatal outcomes occur. Few publications describing the clinical course and outcomes of complicated malaria in Latin America are found in the literature. This prospective study approached the clinical and laboratory characteristics of hospitalized patients with complicated malaria in different endemic areas of the Colombian Pacific Coast with the aim to provide epidemiological knowledge and guide to further reducing malaria severity and mortality.

**Methods and findings:**

A prospective, descriptive hospital-based study was conducted in 323 complicated malaria patients (median age 20 years) enrolled in Quibdó, Tumaco and Cali between 2014 and 2016. Clinical evaluation was performed and laboratory parameters were assessed during hospitalization. *Plasmodium falciparum* was the most common parasite species (70%), followed by *P*. *vivax* (28%), and mixed malaria (*Pf*/*Pv;* 1.9%). Overall, predominant laboratory complications were severe thrombocytopenia (43%), hepatic dysfunction (40%), and severe anaemia (34%). Severe thrombocytopenia was more common in adults (52%) regardless of parasite species. Severe anaemia was the most frequent complication in children ≤10 years (72%) and was most commonly related to *P*. *vivax* infection (p < 0.001); whereas liver dysfunction was more frequent in older patients (54%) with *P*. *falciparum* (p < 0.001). Two deaths due to *P*. *vivax* and *P*. *falciparum* each were registered. Treatment provision before recruitment hindered qPCR confirmation of parasite species in some cases.

**Conclusions:**

The study identified a high prevalence of complicated malaria in the Pacific Coast, together with more frequent severe anaemia in children infected by *P*. *vivax* and hepatic dysfunction in adults with *P*. *falciparum*. Results indicated the need for earlier diagnosis and treatment to prevent complications development as well as more effective attention at hospital level, in order to rapidly identify and appropriately treat these severe clinical conditions. The study describes epidemiological profiles of the study region and identified the most common complications on which clinicians must focus on to prevent mortality.

## Introduction

Malaria-related morbidity and mortality remain important public health problems in the developing world. In 2015, a total of ~212 million malaria clinical cases and ~429,000 related deaths were estimated worldwide [1] most of which were caused by *Plasmodium falciparum* (~90%). Meanwhile, *Plasmodium vivax* was responsible for ~4% of the global cases, 41% of which were reported in regions outside Africa [1]. In 2015, four countries in Latin America were responsible for >80% of *P*. *vivax* cases in this continent: Venezuela (30%), Brazil (24%), Perú (19%), and Colombia (10%) [1–3]. Although Brazil and Colombia reported more malaria-related deaths, with 18 and 37 cases, respectively [1], it is likely that a failure in the recording system explains the lack of fatal cases reported in others countries such as Venezuela.

Malaria clinical spectrum appears to differ depending on the transmission intensity, parasite species, and patient´s immune status [4–6]. Malaria-related deaths, especially due to cerebral malaria and severe anaemia, are more common in children and pregnant women from Africa where there is high malaria transmission intensity and *P*. *falciparum* is the predominant parasite species [7–10]. Severe anaemia and acute renal dysfunction are more frequently reported in *P*. *vivax* endemic settings with significantly lower mortality [11,12]. In Colombia, previous studies have indicated severe anaemia, and hepatic and renal dysfunction as predominant malaria complications, which are distributed in all ages with approximately equal participation of *P*. *vivax* and *P*. *falciparum*, and low associated mortality [4,13–15].

Although Colombia is among the major malaria contributor in Latin America, and has experienced a significant malaria decrease since 2000, complicated cases and malaria-related mortality appear to remain stable, indicating the need for more attention to these issues. Among some studies describing the clinical profile and laboratory parameters in complicated malaria, as well as predictors of life-threatening malaria in this population [4,13,14,16–24], several are retrospective and therefore, potentially biased by the lack of a harmonized protocol. Additionally, associations between *Plasmodium* species and malaria clinical profiles have been hardly explored [4,14,16,22,24].

A recent survey of complicated malaria cases reported to the Colombian Public Health Surveillance System (SIVIGILA) during the 2007–2013 period indicated a total of 547,542 malaria clinical cases, of which 2,553 (0.47%) corresponded to complicated cases leading to 116 malaria-related deaths (0.02%) with similar distribution between *P*. *vivax* and *P*. *falciparum* species [14]. Although the country displayed a decreasing trend in malaria prevalence during the study period, a non-significant annual increase of complicated cases was recorded, with higher frequency of complicated cases and greater mortality in mixed malaria patients (*P*. *falciparum* and *P*. *vivax*); in whom neurological and hepatic manifestations (impaired consciousness, clinical jaundice, and hepatomegaly) were more common. Moreover, a recent retrospective paired, case–control study (2009–2013) which included 159 clinical records of complicated and non-complicated malaria cases described elevated transaminases (44%), clinical jaundice (37%), elevated bilirubin (25%), and thrombocytopenia (20%) as the main laboratory changes associated with severity; whereas severe anaemia was only found in 8% of the cases [25]. However, these two studies may present limitations inherent to retrospective studies such as probable misclassification of complicated malaria cases, errors in species diagnosis, i.e. lack of *Plasmodium* species confirmation by molecular techniques (to ascertain mono-infections or mixed infections), and lack of reliable laboratory data.

Herein, a prospective hospital-based study approached the clinical and laboratory characteristics of hospitalized patients of all ages with complicated malaria. It included patients from endemic areas of Colombia with different epidemiological profiles and transmission of both P. vivax and P. falciparum, in order to generate knowledge to contribute to designing new strategies and providing guidance to further reduce malaria severity and mortality.

## Methods

### Study design

A prospective, descriptive hospital-based study of complicated malaria patients was conducted between 2014 and 2016 in three hospitals located on the Colombian Pacific Coast. The recruitment of patients started on November 2014 in Tumaco, on January 2015 in Quibdó and Cali, and finished on August 2016 in all settings. A total of 323 patients with confirmed malaria infection and one or more criteria of complication as established by World Health Organization (WHO) [26] and the Colombian Minister of Health (MoH) guidelines [27] were enrolled (Table 1). Study physicians and nurse assistants performed an active search of complicated malaria patients daily at study hospitals. Once the complication criteria were confirmed, before enrolment, patients were asked to participate in the study and provide an informed consent/assent (IC/IA), previously approved by the Ethics Committee. Blood samples were taken by the nurse assistant and the medical evaluation was performed by the study physicians.

**Table 1:** Severe malaria criteria according to WHO [26] and the Colombian MoH guidelines [27].

Criterion	Description
Cerebral malaria	Coma, Blantyre coma score < 3 or Glasgow score < 9
Inability to oral intake	Inability to eat and/or drink due to persisting vomiting or extreme weakness
Prostration	Generalized weakness where patient is unable to walk or sit up without assistance
Multiple convulsions	More than two episodes in 24 h
Respiratory distress	Presence of alar flaring, chest recession or abnormal deep or acidotic breathing
Pulmonary oedema	Confirmed by Chest X-Ray
Circulatory collapse or shock	Systolic blood pressure <70 mm Hg in adults or <50 mm Hg in children (3–5 years)
Abnormal spontaneous bleeding	Bleeding from the nose, venipuncture sites, gums, or gastrointestinal tract in the presence of laboratory evidence of disseminated intravascular coagulation (DIC)
Hyperpyrexia	Fever >40°C that persists after initiation of antimalarial treatment
Severe thrombocytopenia	<50,000 platelets/μL
Haemoglobinuria	Macroscopic and positive urine dipstick, in absence of microscopic haematuria
Severe anaemia	Hb <7 g/dL [27]
Hepatic dysfunction	Serum bilirubin >3 mg/dL or ALT/AST >120 U/L
Renal dysfunction	Serum creatinine >1.5 mg/dL [27] or BUN >40 mg/dL
Hypoglycaemia	Blood glucose level <60 mg/dL
Hyperparasitaemia	>50,000 asexual parasites/μL [27]
Acidosis	Serum HCO3 <15 mmol/L or Base Excess >-10 on arterial blood gases.

A case report form (CRF) was designed to record patient information by the study physician directly from the patient. This information included: demographic data (age, sex, name, occupation, education grade, site of origin), past medical history (previous malaria episodes, comorbidities), clinical information of the current episode (symptoms, days of disease), medical evaluation (vital signs, conscious scale, a complete physical examination), if transfusions were performed and finally the clinical outcome and discharge diagnosis. These data were added to the patient’s medical records. No data were taken from the hospital records.

The medical examination was performed during enrolment and included the assessment of vital signs, conscious scale and a complete physical examination by systems. In addition, patients were followed daily by the physicians for clinical evolution at hospital until they were discharged.

### Ethics statement

The study protocol was reviewed and approved by the institutional Review Board (IRB) named Ethics Committee of the Centro Internacional de Vacunas (CECIV, Cali-Colombia) before initiation of patient’s enrolment. Written IC was obtained from each volunteer at enrolment. Parents or legal guardians were asked to consent for children (<18-year-old) to participate in the study. Additionally, children older than seven years were asked to sign an informed assent to ensure their willingness to participate. A trained physician of the study team completed a standard clinical evaluation form of all subjects. Nurse assistants were responsible for the blood sample collection. Information obtained from the participants was managed on principles of confidentiality. Immediately after blood sample processing, malaria-positive volunteers were informed and assessed during administration of appropriate anti-malarial treatment at the corresponding point of care.

### Study sites

**Fig 1:**
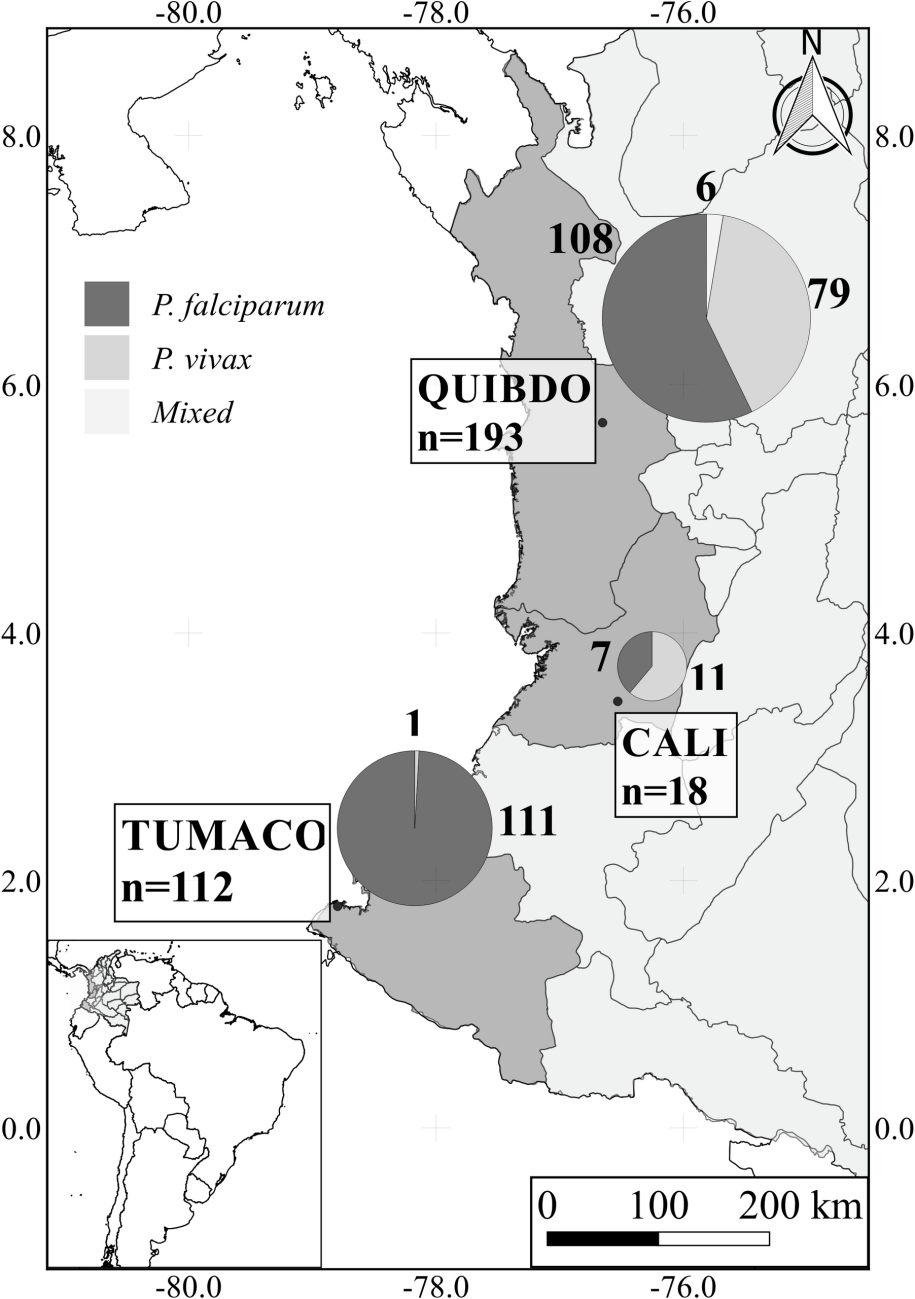
Distribution of complicated malaria cases in the study areas. For each of the three study sites, the total number of complicated malaria cases is shown on the map, as well as a pie chart with the proportion of *Plasmodium* species per site. *Plasmodium vivax* cases were more frequent in Cali (61%), whereas *P*. *falciparum* infections were more common in Quibdó (56%) and Tumaco (99%).

The study was conducted in two hospitals located in a malaria endemic area of the Colombian Pacific Coast: San Francisco de Asís Hospital (level II) located in Quibdó (Capital of Department of Chocó) and San Andres (level II) Hospital located in Tumaco (Department of Nariño). Quibdó is located on northwest region in proximity to Panamá, and Tumaco is located on southwest region of the Pacific Coast in proximity to Ecuador (Fig 1), both areas considered of moderate-to-high malaria transmission intensity with presence of both *P*. *vivax* and *P*. *falciparum* species. Average annual parasite index (API) between 2011 and 2013 in Quibdó and Tumaco were 25 and 10.3 respectively. The third hospital was the University Hospital Evaristo Garcia (level III), located in Cali (Department of Valle del Cauca), a city without malaria transmission, which was included because complicated malaria patients are regularly referred from multiple endemic municipalities of the Pacific Coast region.

Quibdó has a total population of ~115,000 inhabitants, predominantly afro-descendants (95.3%) and indigenous (1.4%), corresponding to ~23% of the Choco population (500,000 inhabitants) [28]). In 2016, from the ~83,000 total malaria cases reported in Colombia, 56.5% (~47,000) originated in Choco, and 16.2% (~13,200) were registered in Quibdo. Most of the cases were reported in Choco (46,202; ~98%), consisting mainly of P. falciparum (67.7%), and were non-complicated malaria, whereas the remaining ~700 were complicated cases. As with the non-complicated cases, the latter cases corresponded to approximately half of the total complicated cases registered in the whole country [29]. A recent study indicates that although most cases in Quibdó were reported from urban Points of Care (POC), virtually all malaria cases in Quibdó were generated in rural and peri-urban areas [30]. Tumaco has a total population of ~203,000 inhabitants, afro-descendants (~19%) and indigenous (~11%), corresponding to ~11.7% of the Nariño population (~1,740.000 inhabitants) [28]. In 2016, Nariño was the second region with most malaria cases in the country (10,715 cases, 13.1%) after Chocó. Tumaco reported 4.1% of the total malaria cases in the country (~3,300 cases) with P. falciparum as the main parasite species (~97.2%) and around 8.3% (124/1,494) of complicated malaria cases in 2016 [29]. **Cali**, with a total population of ~2.7 million, is located in the southwest of the country with no malaria transmission, however the Department of Valle del Cauca reported ~4% of complicated malaria cases from patients referred from adjacent malaria endemic areas (mainly Chocó) in the same years, most of them due to *P*. *falciparum* [29]. During 2016, around 37% of complicated malaria cases in Colombia occurred in population between 15–30 years of age. [29]

### Case definition

A complicated malaria case was defined by clinical malaria manifestations, i.e. history of fever and a positive thick blood smear (TBS) performed at local hospital, plus the presence of one or more clinical and/or laboratory parameters as established by WHO [26] and the Colombian MoH guidelines [27] (Table 1). The latter are more conservative in some definitions based on previous evidence: severe anaemia (Hb <7g/dL), renal dysfunction (serum creatinine >1.5mg/dL), severe thrombocytopenia (≤50,000 platelets/μL), and hyperparasitaemia (>50,000 parasites/μL). The pregnancy status was confirmed either by pregnancy dipstick test, clinical evaluation, and/or ultrasound report. The gestational age was measured calculating the days from the beginning of the last menstrual period and/or the earliest ultrasound. All patients of any age who met case definition criteria, gave IC/IA, and did not present any renal, pulmonary, or hepatic chronic conditions, were enrolled.

Patients with malaria-compatible symptoms seek medical attention at peripheral POC, where malaria diagnosis is performed either by microscopy or rapid diagnostic tests (RDT) and non-complicated patients are provided with free-of-charge treatment. Complicated cases are either referred from the POC or consult directly to the local hospitals, usually second level of complexity. Patients with danger signs or complication criteria as established by the MoH and/or WHO (i.e. anaemia, thrombocytopenia, clinical deterioration and others) are referred to higher complexity hospitals at the study settings.

### Laboratory tests

After informed/assent consent, whole blood (27 mL from adults and 18 mL from children <10 years of age) was collected by venipuncture at the time of enrolment once the malaria diagnosis was confirmed by microscopic examination of Giemsa-stained TBS. Parasite density (parasites/μL) was estimated by counting the number of parasites per 200 leukocytes and normalized using the current leukocyte count of each patient [(number of parasites x leukocyte counts)/200 leukocytes]. Prior to study initiation, laboratory tests such as automated complete blood cell count, biochemistry profile (creatinine, blood urea nitrogen, total bilirubin, aminotransferases, and glycaemia), and diagnostic tests (TBS and qPCR) were standardized to comply with similar SOPs, to ensure similar performance in the different laboratories. Laboratory technicians were trained to perform similar procedures.

As quality control, a second analysis of the thick smears was performed by an independently trained malaria microscopist. In the case of discordant results, the slides were read by a third microscopist. The definitive result was one of which at least two reads matched. The quantitative PCR (qPCR) [4] was performed with blood samples collected at enrolment and malaria parasite species was retrospectively confirmed in all samples. Automated complete blood cell count, renal function (creatinine, blood urea nitrogen), hepatic function (total bilirubin and aminotransferases: ALT and AST), and glycaemia were performed at each hospital for most patients and blood cultures were performed to discard concomitant bacterial infections.

### Statistical analysis

The main objective of this study was to find associations between age groups (children and adults), the malaria parasite species (P. vivax and P. falciparum) and the type and severity of the clinical complication(s). Therefore, outcomes measured were: parasitemia, haematological and blood chemistry parameters as well as clinical manifestation in patients infected with either P. vivax or P. falciparum. Categorical variables were summarized as frequency (proportion) of complicated malaria cases by P. vivax or P. falciparum species of each study sites. Chi-square or Fisher´s exact tests were used for comparison of categorical data: clinical presentation and complication pattern in adults/children and in P. vivax and P. falciparum infections. Patients enrolled (1–84 years old) were divided into two age groups: younger than 10 years old, and 10 and older. The severity of complication was determined by laboratory variables according to the numeric ranges established within the Colombian MOH and/or WHO guidelines for complicated malaria [27,31]. Study data were collected and managed using REDCap (Nashville, Tennessee, USA) with electronic data capture tools [32]. Statistical analysis was performed with R version 3.3.2, 2016 (The R Foundation, Vienna, Austria). Nominal variables were analysed using descriptive statistics. The Mann-Whitney U or Kruskal-Wallis tests were used to compare the different groups when appropriated. Chi-square or Fisher's exact test were used to compare proportion differences. A p value <0.05 was considered statistically significant.

## Results

### Demographic and epidemiological characteristics

A total of 323 hospitalized patients were enrolled: 193 in Quibdó, 112 in Tumaco, and 18 in Cali (Fig 1); with similar proportion between females and males (~50%). *Plasmodium falciparum* was the most frequent species (n = 226; 70.0%), followed by *P*. *vivax* infections (n = 91; 28.2%), and a low number of mixed *Pf/Pv* cases (n = 6; 1.9%) which were all recruited in Quibdó (Fig 1). Ninety-eight (30%) enrolled patients were children between 0 and 10 years of age and 225 (70%) between 11 and 84 years of age. A significantly higher median age was observed in patients infected by *P*. *falciparum* than those with *P*. *vivax* (22 *vs*. 9 years; p < 0.001).

**Fig 2:**
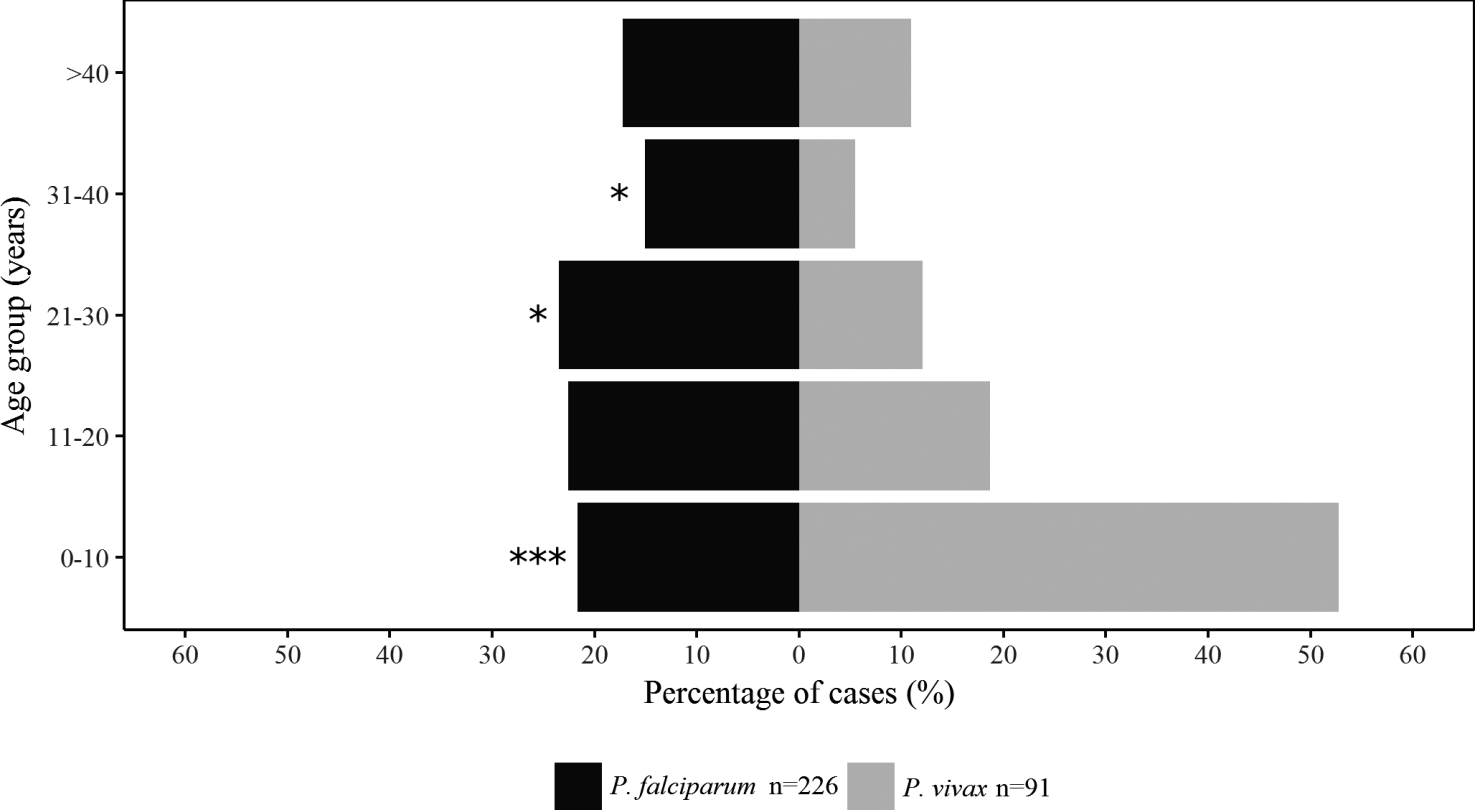
Prevalence and parasite species distribution according to age. Percentage of individuals infected with either *P*. *falciparum* or *P*. *vivax* parasites stratified by age group. Statistical differences between *P*. *falciparum* and *P*. *vivax* infections were calculated using the Chi-square test. *p value < 0.05, **p value < 0.01, *** p value < 0.001.

Most of the *P*. *vivax* patients (48/91) were children between 0–10 years of age, while *P*. *falciparum* cases were more evenly distributed among all age groups (Fig 2). Notably, 30 out of 151 enrolled women were pregnant (20%). Afro-descendant patients (48%) represented the most frequent ethnic group and were mainly infected with *P*. *falciparum* (p < 0.01), whereas most of the patients infected with *P*. *vivax* (41%) were indigenous (p < 0.01). The site of residency was almost equally distributed between rural (47%) and urban (52%) settings. Only 50 patients (15.5%) self-reported previous malaria episodes, and in 24 of them the last episode was in the previous year.

The Colombian MoH adopted more conservative criteria than WHO since 2010 because the latter were highly focused on *P*. *falciparum* in highly endemic areas of Africa, whereas malaria transmission in Colombia is of low to moderate intensity and is caused by both *P*. *falciparum* and *P*. *vivax*, predominantly the latter. Moreover, many complicated cases are referred from remote areas and would benefit from the use of more conservative criteria for hospital referral.

In general, malaria mortality in Colombia is considered low, (~0,02% in 2016) [33]. We believe that this trend is highly influenced by the early consultation of malaria patients in Colombia [34] as well as the use of more conservative criteria to treat severe cases. Thus, the main advantage of the complication criteria established by the Colombian MoH is to cover detection and careful treatment of malaria cases with a higher risk of mortality. Nevertheless, mortality may be the result of late referral of complicated cases from peripheral low level hospitals to better equipped third and fourth level hospitals usually located in mains cities [35].

Analyses were done to evaluate the adequacy of the severe malaria criteria for the specific Colombian settings. The group of patients with severe anaemia, severe thrombocytopenia and hepatic dysfunction was analysed by using both the MoH and the WHO criteria (Table 2). Mean duration of hospitalization and requirement of ICU were assessed. No significant differences were found in terms of duration of hospitalization and ICU requirement between patients with severe anaemia and hepatic failure groups, however, a higher proportion of patients with severe thrombocytopenia as per WHO parameters required ICU than those following MoH criteria (16.7% vs 6.4%, respectively).

**Table 2:** Mean hospitalizations days and number of patients at the ICU.

Parameter	Criteria	n	Mean hospitalization days	Patients at ICU Number (%)
**Severe anaemia**	MoH Hb < 7g/dL	111	5.83	4 (3.60)
	WHO Hb < 7g/dL in adults and Hb < 5g/dL in children	73	6.00	2 (2.74)
**Hepatic dysfunction**	MoH serum bilirubin: >3 mg/dL or ALT/AST: >120 U/L	131	5.27	7 (5.34)
	WHO serum bilirubin: >3 mg/dL only	88	5.43	5 (5.68)
**Severe thrombocytopenia**	MoH <50,000 platelets/μL	140	5.00	9 (6.43)
	WHO <20,000 platelets/μL	12	5.58	2 (16.67)

Analyses of patients with 1 and those with >1 malaria severity criteria including days of hospitalisation, ICU management, haemoglobin levels, platelets count, parasitemia, glucose levels, renal and hepatic function as surrogate markers indicated no significant differences in terms of days of hospitalization between the two groups, however, patients with 2 or more criteria required ICU management more frequently than those with only one (0.7% vs 7.0%). In terms of laboratory parameters, significantly worse values were found for platelet levels, hepatic and renal function in patients with >1 complication criteria (Table 3).

**Table 3:** Laboratory differences in patients with more than one severity criterion.

Parameter	n	Median of group with 1 criterion	Median of group with >1 criterion	P value
**Haemoglobin (g/dL)**	323	8.6	9.6	<0.05
**Platelets (x 103/μL)**	323	72	43	<0.001
**Bilirrubin (mg/dL)**	186	1.34	3.5	<0.001
**AST (U/L)**	218	51	102	<0.001
**ALT (U/L)**	218	40	111.5	<0.001
**Creatinine (mg/dL)**	265	0.8	1	<0.001
**BUN (mg/dL)**	194	13	16	<0.01
**Parasitaemia (Parasites/μL)**	303	5800	8040	0.099
**Glucose (mg/dL)**	206	96	99	0.548
**Leucocytes (x 103/μL)**	322	4.8	4.83	0.730

In addition, analyses to compare the surrogate markers of severity (days of hospitalization, ICU management, haemoglobin levels, platelets count, parasitemia, glucose levels, renal and hepatic function parameters) between patients with 5 and > 5 days of disease (time between symptoms onset and recruitment day) were performed. However, no significant associations were found on any of the evaluated parameters between groups. Therefore, in this study a longer duration of disease was not associated with greater severity of the malaria episode.

In this study, only 50 patients reported previous malaria episodes and this was not related with the severity degree (number of parameters) during the present illness.

### Malaria diagnosis

Most patients (69%) presented with low-to-moderate parasitaemia (≤20,000 parasites/μL), with a median of 5,220 parasites/μL (IQR 1,760–22,080) in infections by *P*. *falciparum* and 9,050 parasites/μL (IQR 4,305–17,620.5) in *P*. *vivax* (Table 4). However, 27 out of the 34 patients presenting hyperparasitaemia (>50,000 parasites/μL) were infected by *P*. *falciparum*. No significant differences on parasite densities were observed between age groups. Although qPCR was retrospectively done for every single patient at enrolment, 213 (42.7%) were negative, presumably due to the treatment initiated upon consultation to the POC, since every referred patient presented a positive TBS. Treatment of these patients consisted of chloroquine (25mg/kg divided in three doses) + primaquine (0.25mg/kg/day for 14 days) for most of *P*. *vivax* cases. Artemether + lumefantrine (20mg + 120mg in adults for six doses and according to weight and age in children < 34kg and/or <14 years of age) for most of *P*. *falciparum* cases. Artesunate and Quinine were also used in some cases. No specific characteristics distinguished them from other patients with positive qPCR.

Overall, patients attended late for diagnosis, most (78.2%) of them ≥72h after symptoms onset (IQR 3–7 days). No significant differences were found in terms of days of illness or hospitalization time between *Plasmodium* species.

**Table 4:** Laboratory parameters in children and adults with complicated malaria according to *Plasmodium* species.

Laboratory parameter	*P*. *falciparum* n = 226 Median (IQR^*a*^)				*P*. *vivax* n = 91 Median (IQR^*a*^)				*Pf vs*. *Pv*
	Children (<10 y) n = 49	Adults (>10 y) n = 177	All	p value^*b*^	Children n = 48	Adults n = 43	All	p value^*b*^	p value^*c*^
**Parasitaemia**	4,560 (1,560–16,640)	5,295 (1,802–24,000)	5,220 (1,760–22,080)	0.487	10,280 (3,670–21,450)	8,571 (5,000–12,880)	9,050 (4,305–17,620)	0.469	0.126
**Haematological test**									
**Haemoglobin (g/dL)**	6.0 (3.9–8.6)	10.5 (8.3–12.0)	9.9 (6.6–11.7)	<0.001	5.4(4.4–6.8)	10.0 (8.2–11.9)	7.2 (5.0–10.1)	<0.001	<0.001
**Leukocytes (x 10**^**3**^**/ μL)**	6.7 (4.7–9.2)	4.4 (3.5–5.9)	4.7 (3.6–6.3)	<0.001	5.8 (3.9–8.2)	4.9 (3.5–6.5)	5.1 (3.7–7.1)	0.091	0.175
**Platelets (x 10**^**3**^**/ μL)**	95.0 (58.0–177.0)	54.0 (37.0–79.0)	58.0 (39.0–96.8)	<0.001	68.0 (44.7–102.0)	38.2 (30.7–59.0)	52.0 (34.0–84.5)	<0.001	0.165
**Biochemical test**									
**Total bilirubin (mg/dL)**	1.4 (0.7–3.9)	3.4 (1.3–5.7)	3.2 (1.2–5.5)	0.03	1.0 (0.7–1.2)	2.5 (1.1–4.6)	1.7 (0.9–3.4)	0.006	0.037
**ALT (U/L)**	27.0 (20.5–120.5)	109.0 (43.8–181.8)	102.0 (33.5–175.5)	0.003	21.0 (13.0–31.0)	55.0 (26.0–142.5)	31.0 (18.0–80.0)	0.0014	<0.001
**AST (U/L)**	50.0 (32.5–207.5)	110.50 (51.0–166.7)	99.0 (40.5–169.5)	0.092	35.0 (26.0–53.0)	53.0 (34.8–82.3)	43.0 (29.0–75.0)	0.023	<0.001
**Creatinine (mg/dL)**	0.5 (0.4–0.6)	1.10 (0.9–1.6)	0.9 (0.7–1.4)	<0.001	0.5 (0.4–0.6)	1.10 (1.0–1.4)	0.8 (0.5–1.1)	<0.001	0.0026
**BUN (mg/dL)**	11.0 (8.0–13.0)	16.0 (11.0–27.0)	13.0 (9.9–23.9)	<0.001	11.0 (7.0–15.0)	17.2 (13.0–25.0)	15.0 (10.0–19.8)	<0.001	0.566
**Glucose (mg/dL)**	96.0 (88.5–111.7)	93.5 (78.3–106.8)	94.0 (79.0–109.1)	0.251	110.0 (89.0–121.0)	105.3 (91.8–121.8)	106.0 (90.0–121.0)	0.891	0.0036

^*a*^ IQR: interquartile range.

Abbreviations: ALT: Alanine aminotransferase; AST: aspartate aminotransferase; BUN: Blood urine nitrogen.

^b^ p value using Mann-Whitney test between children and adults.

^*c*^ p value using Mann-Whitney test between *P*. *falciparum* and *P*. *vivax*.

### Clinical findings and complications

**Fig 3:**
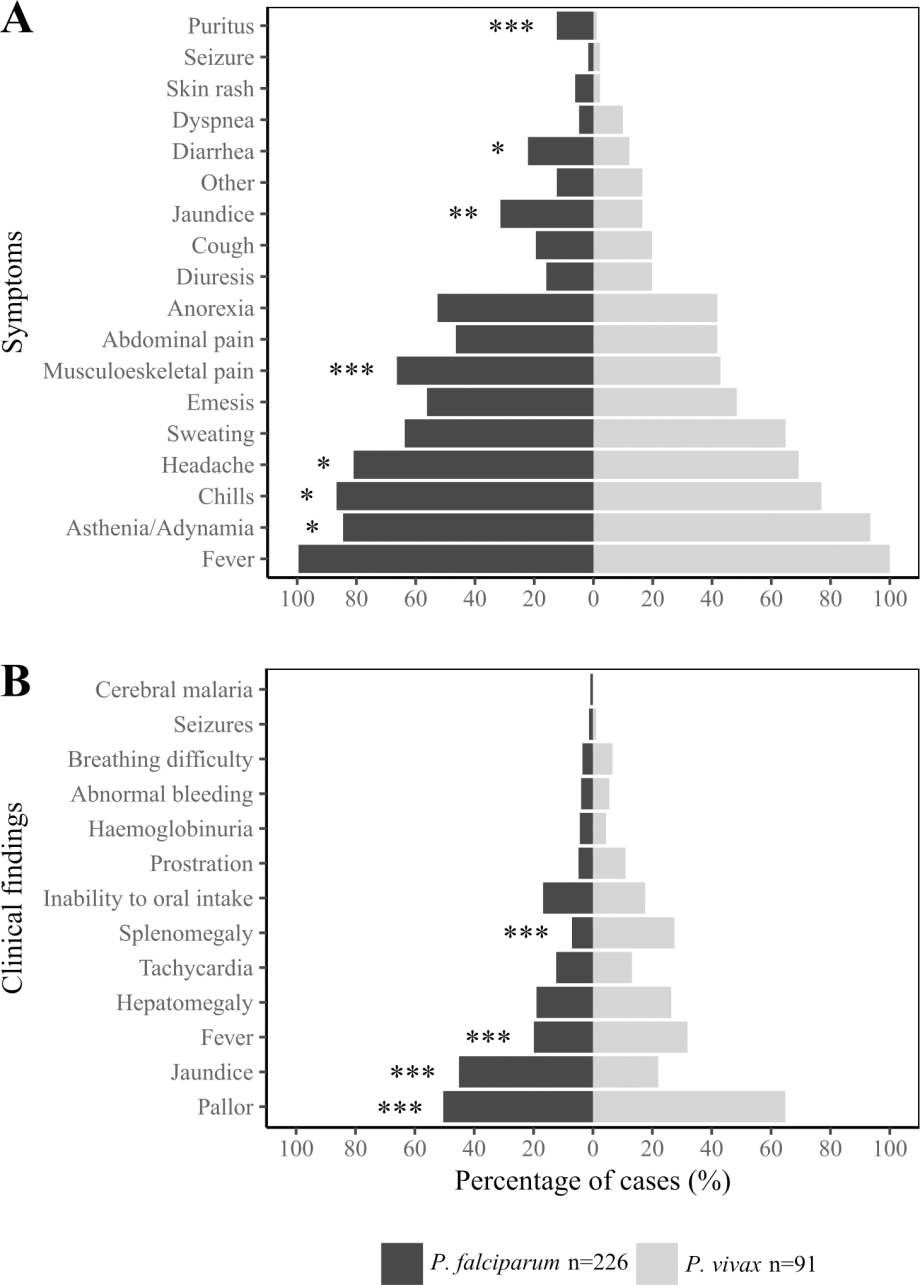
Frequency of clinical manifestations in *P*. *falciparum* and *P*. *vivax* infections. Percentages of malaria patients that reported every symptom (A) or presented with the listed clinical findings (B) are shown. All patients reported more than one symptom or had more than one sign. Statistical differences between species were calculated using the Chi-square test. *p value < 0.05, **p value < 0.01, *** p value < 0.001.

Fever, asthenia/adynamia and chills were the most common symptoms among complicated malaria patients. The frequency of certain symptoms and clinical findings was different between *Plasmodium* species. In *P*. *falciparum* cases, headache (p = 0.03), musculoskeletal pain (p *=* 0.0001), jaundice (p *=* 0.0008) and probable jaundice-related pruritus (p *=* 0.001) were the more frequent, whereas *P*. *vivax* patients presented significantly more asthenia/adynamia (p *=* 0.04) (Fig 3A). At physical examination, jaundice was a common finding in *P*. *falciparum*, whereas pallor and hepato-splenomegaly were more frequent in *P*. *vivax* (p <0.001) (Fig 3B).

A total of 612 complications were recorded among the 323 enrolled patients, with 170 presenting two or more complication criteria (range 2–8). Clinical complications were less common than laboratory complications (130/612; 21% *vs*. 482/612; 79%, respectively). The most frequent clinical complications were inability to oral intake (16%), prostration (7%) and abnormal bleeding (5%). Prostration was significantly more frequent in children ≤10 years of age than in older patients (12% *vs*. 4%, respectively: p *=* 0.012).

### Laboratory alterations

**Fig 4:**
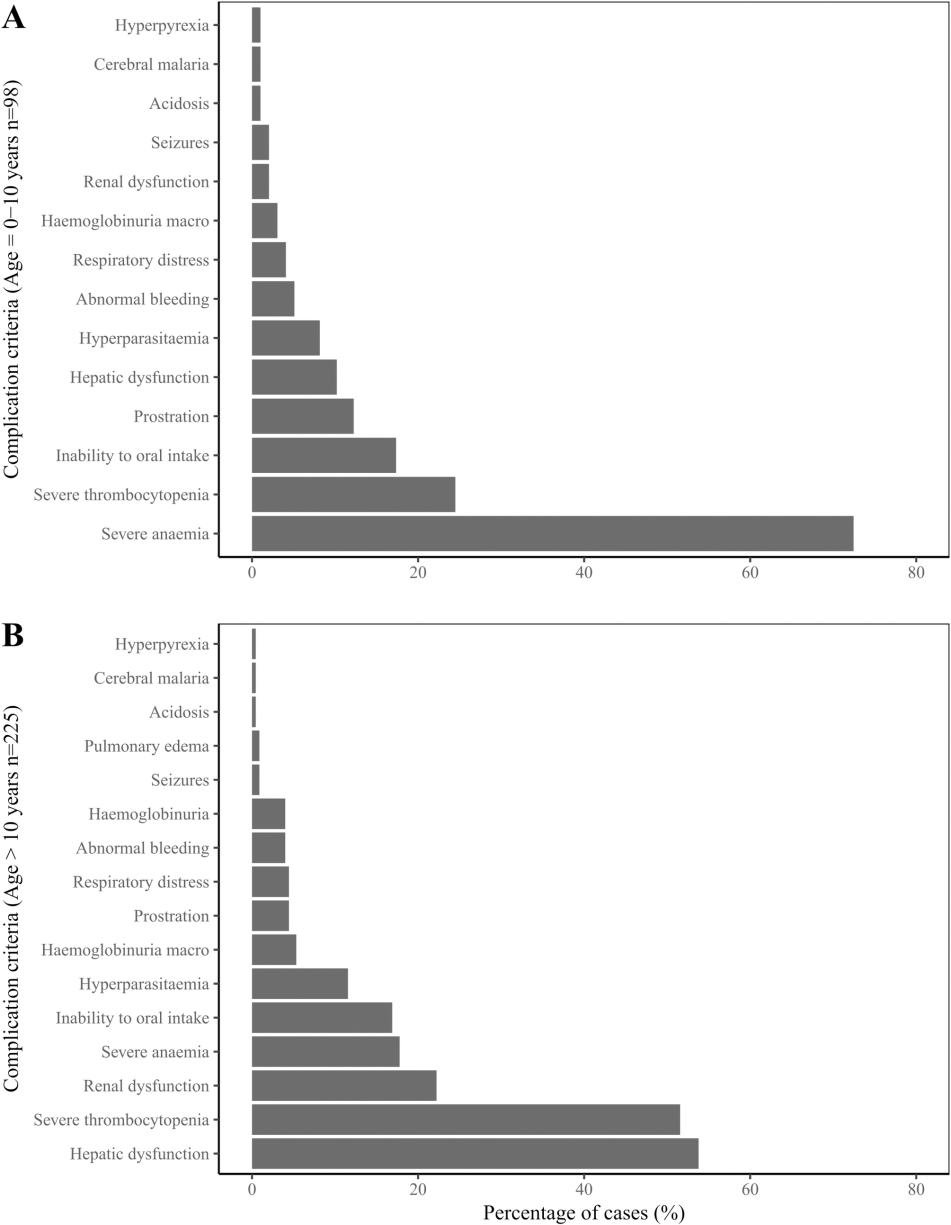
Frequency of complications by age group. Percentage of malaria patients that presented each of the indicated WHO/MoH complication laboratory criterion are shown for children ≤10 years of age (A) and individuals >10 years (B).

Overall, 43% of patients presented severe thrombocytopenia (<50,000 platelets/μL), 40% hepatic dysfunction (total bilirubin >3.0 mg/dL or AST or ALT >120 U/L), and 34% severe anaemia (Hb <7.0 g/dL). The most common laboratory complication in children ≤10 years of age was severe anaemia (n = 71/98, 72%), whereas in patients older than 10 years of age it was hepatic dysfunction (n = 121/225, 54%), mainly due to *P*. *falciparum* infection (Fig 4). The majority of patients presented more than one clinical and/or laboratory complication (170/323; 53%). The most common associations for laboratory complications were severe thrombocytopenia/hepatic dysfunction (54%), followed by severe thrombocytopenia/renal dysfunction (21%), regardless of parasite species.

#### Haematological parameters

**Fig 5:**
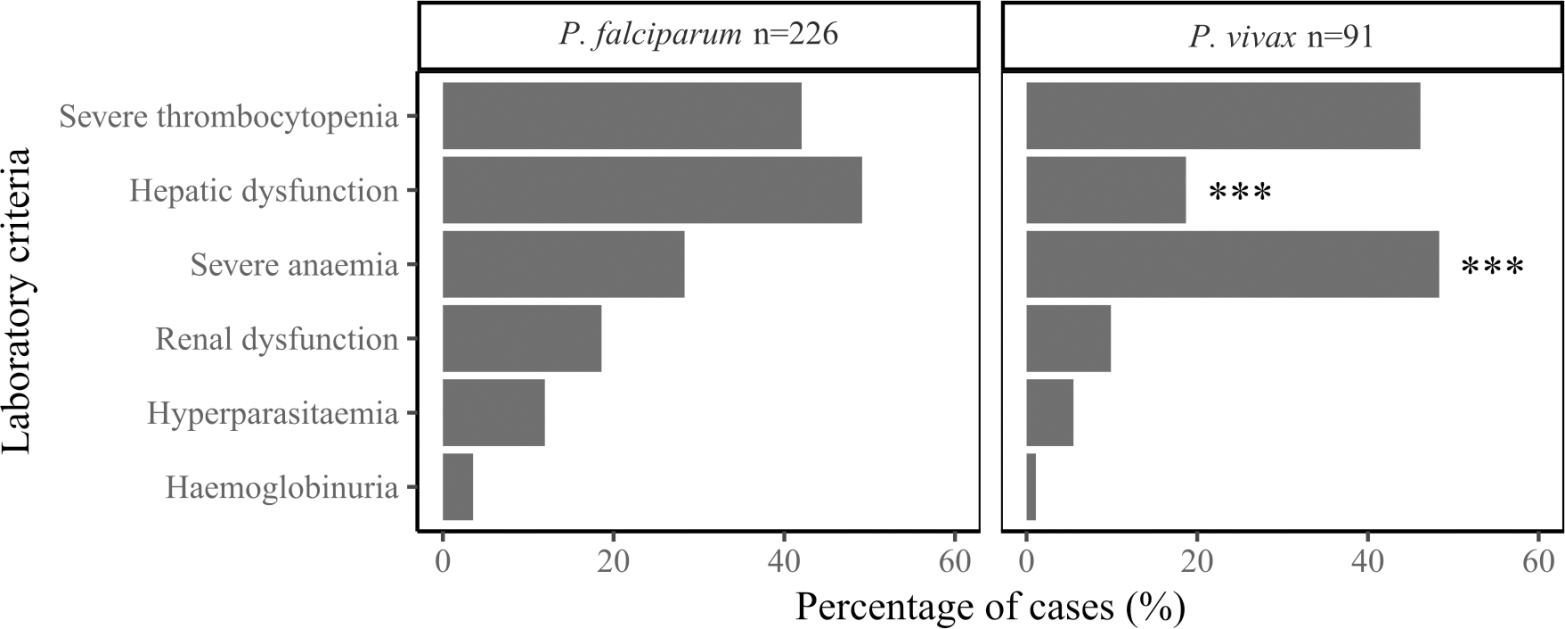
Frequency of laboratory complications in *P*. *falciparum* and *P*. *vivax* cases. Percentage of malaria patients that presented each of the indicated WHO/MoH complicated laboratory criterion are shown for each *Plasmodium* species.

Most complicated malaria patients (282/323, 88%) were thrombocytopenic (<150,000 platelets/μL) and 81% (264/323) were anaemic (Hb <12 g/dL), with lower Hb levels in *P*. *vivax* than in *P*. *falciparum* cases (Table 2). However, no significant differences in platelets counts were found between parasite species. Severe anaemia was significantly more frequent in *P*. *vivax* than in *P*. *falciparum* cases (48% *vs*. 28%, respectively; Fig 5).

Of the 323 patients with complicated malaria, 121 (37.5%) required blood transfusion, 50 with Hb <5.0g/dL, 53 with Hb <7.0 g/dL and ≥5.0 g/dL and the remaining 18 with Hb <12.0 g/dL and ≥7.0 g/dL and blood cultures were performed in 137 patients. Only one patient presented a bacteraemia by *Salmonella spp*, 112 blood cultures were either negative (81.7%) and 25 (18.2%) were positive for *Staphylococcus epidemidis* among others, probably due to accidental contamination.

#### Hepatic and renal function parameters

Hepatic function was abnormal in most patients, as 147/186 (79%) presented elevated bilirubin levels (>1 mg/dL) and 169/226 (78%) elevated aminotransferases AST or ALT levels (>39 U/L). No associations between indirect bilirubin levels and haemoglobin levels were found among patients with hyperbilirubinemia. Renal function as per creatinine (>1.5 mg/dL) and BUN (>40 mg/dL) levels was altered in a low percentage of cases (48/265, 18% and 9/194, 5%, respectively).

### Pregnant women

A total of 30 women were pregnant, mainly from Quibdó (n = 26, 87%) and infected by *P*. *falciparum* (n = 24, 80%). Their median age was 22 years (range 11–43 years) and the median gestational age was 30 weeks (range 10–40 weeks). Eight were primigravidae and the median parasitaemia was 4,800 parasites/μL for *P*. *vivax* (range 400–20,800 parasites/μL) and 12,000 parasites/μL for *P*. *falciparum* (range 560–125,000 parasites/μL). The main complication was severe anaemia (n = 16, 53%), followed by severe thrombocytopenia (n = 9, 30%), and hyperparasitaemia (n = 6, 20%). Only one 20-year-old patient with mixed infection, first pregnancy, and unknown gestational age, presented an abortion. Most patients were discharged before delivery in good clinical conditions (n = 25, 83%). Three gave birth during the hospitalization and one of them underwent a caesarean section.

### Malaria related-deaths

Two patients from Quibdó died during the study period. One was a 4-year-old female child who presented cerebral malaria, history of multiple seizures, severe anaemia, thrombocytopenia, and hyperparasitaemia due to *P*. *falciparum*, who died <72 hours after admission. The other patient was a 19-year-old male with *P*. *vivax* malaria who presented neurological impairment, hepatic dysfunction, severe thrombocytopenia and a HIV co-infection diagnosed during hospitalization. He presented progressive neurological decline and persistence of fever despite antimalarial treatment. The death occurred 13 days after hospital admission and was probably related to a central nervous system opportunistic infection. Although cerebrospinal fluid examination was not performed, the blood culture was negative.

## Discussion

During a two year-long period a total of 305 complicated malaria cases were recorded in two malaria endemic areas of Colombia: Tumaco and Quibdó. Eighteen cases were enrolled in Cali, a non-endemic city that usually serves malaria patients who are referred from Buenaventura and Quibdó. Important associations were found between the complication parameters, the *Plasmodium* species and the age range of complicated malaria patients. Most patients were adults presenting with *P*. *falciparum* malaria and moderate parasite counts. Severe thrombocytopenia was the most common complication overall and was significantly more common in patients older than 10 years of age. Severe anaemia was predominantly found in children, who were mostly infected by *P*. *vivax*, and hepatic dysfunction was the main complication in adults presenting *P*. *falciparum* infections. Two deaths due to *P*. *vivax* and *P*. *falciparum* each were registered, both with multiples complication including cerebral malaria.

Because the recruitment of complicated malaria patients was performed at the best-equipped hospitals in each region, and strictly following the WHO and MoH guidelines, it appears this led to discordant results with the official SIVIGILA records. In 2015, SIVIGILA recorded total of 190 complicated malaria cases in the study region, 114 in Quibdó, 56 in Tumaco and 20 in Cali, whereas we recorded 162 complicated cases (101 in Quibdó, 51 in Tumaco and 10 in Cali) [36]. This ~15% difference may be explained by several factors including the referral of some patients to other hospitals, a non-strict attachment to the guidelines, and possibly other reasons. Overall, during 2015 and 2016 Chocó department reported 74,484 malaria cases corresponding to 51% of the total malaria cases in the country [29,37,38]. This explains why in this study most of the complicated malaria cases (n = 193 cases) were enrolled in Quibdó, the capital of Chocó, which reported around 35% of the total malaria cases in the department over 2015 (n = 114/326) [29,36]. It is highly likely that numerous cases within the remaining 65% malaria patients in the department corresponded to complicated patients that were included in the present study. In terms of mortality, SIVIGILA reported 60 cases in the whole country between 2014 and 2016, 41 of them from Chocó and Nariño. This study found that only two malaria-related deaths occurred in Quibdó during 2015 and 2016, whereas SIVIGILA reports six death cases in the study settings in 2015. It is probable that some deaths corresponded to patients that did not visit the study hospitals.

Although in the present study, only 28.2% of cases were caused by *P*. *vivax*, which is in contrast with previous Colombian reports in which the prevalence of this species ranged between 44–76% [4,14,24], it is important to highlight that the present study cases were enrolled mainly in Quibdó and Tumaco, where *P*. *falciparum* is the most frequent parasite species. Despite *P*. *vivax* was for many years the predominant malaria species in Colombia, an increase in total malaria cases together with *P*. *falciparum* malaria prevalence have been reported over the last two years, from 18,642 of *P*. *vivax* and 18,343 of *P*. *falciparum* cases (45%) in 2014 [37] to 33,055 and 47,497 cases (57%) in 2016, respectively [29].

In this series, most patients (75%) presented with moderate parasitaemia (< 20,000 parasites/μL) and a higher median parasitaemia was found in *P*. *vivax* than in *P*. *falciparum*, similar to what has been reported in other sites of Colombia (Medellin and Risaralda) [16,24]. Although it has been classically stated that *P*. *falciparum* produces higher parasitaemias than *P*. *vivax*, as the latter only invades immature erythrocytes; higher mean parasite counts have also been reported in vivax than in falciparum complicated malaria patients in Sudan (5,934 *vs*. 13,907 parasites/μL, p = 0.013, respectively) [39]. However, very high parasitaemias are almost exclusive of *P*. *falciparum*, as in this study where 87% of patients with hyperparasitaemia (>50,000 parasites/μL) were infected by this parasite species.

Thrombocytopenia was the most common laboratory complication but no significant differences were found between *Plasmodium* species. Almost 90% of cases presented thrombocytopenia of any grade (<150,000 platelets/μL) with 43% of these corresponding to severe thrombocytopenia (<50,000/μL) cases. In contrasts to these study findings, there is evidence that thrombocytopenia is more frequent in *P*. *vivax*, than *P*. *falciparum* patients [40], although a consensus does not exist [41]. In some endemic areas, malaria has been reported as the major cause of low platelet counts, and it is used as an indicator of malaria in patients presenting with fever, increasing the likelihood of malaria 12–15 times [42]. However, the fact that dengue usually presents with a similar clinical picture (fever and thrombocytopenia), makes mandatory to perform laboratory confirmation of malaria infection in dengue-endemic regions such as Colombia. Moreover, regardless of it being described as a complication by WHO, thrombocytopenia is not considered a severity criterion by itself, as risk of abnormal bleeding is below 10% and no fatal malaria cases with thrombocytopenia as the only complication have been described [43,44].

We found that *P*. *vivax* complicated malaria was more common in children under 10 years of age. This is higher than reported in a systematic review of the Brazilian literature where *P*. *vivax* complicated malaria was recorded in all age groups, with 25% of *P*. *vivax* cases affecting children 0–14 years of age [44]. In Southeast Asia *P*. *vivax* is considered to be a disease of children (< 9 years) because acquisition of immunity to this species occurs much faster than for *P*. *falciparum* in highly endemic areas [45].

About 80% of patients presented any grade anaemia (Hb <12 g/dL) and in 34% it was severe (Hb <7 g/dL); this is in agreement with previous studies in Colombia where anaemia prevalence in complicated malaria cases ranged between 75 and 92%. However, severe anaemia has been reported in up to 52% of cases [13,16,18]. Additionally, severe anaemia was the main complication in children and pregnant women as reported elsewhere [11,46,47], and significantly more common in *P*. *vivax* than in *P*. *falciparum* cases (Fig 5). Indeed, *P*. *vivax* has been considered a major risk factor for severe anaemia in vivax-endemic areas of Papua and Papua New Guinea, particularly in young children [11,47].

Interestingly, Chaparro-Narvaez *et al* reported that hepatic and pulmonary compromise were significantly more frequent in *P*. *vivax*, whereas cerebral malaria and renal dysfunction were present in *P*. *falciparum* infections regardless of age [14]. On the other hand, in the current study most complicated malaria cases presented abnormal hepatic function parameters (bilirubin and aminotransferases levels), which indicates liver damage, and this was significantly more frequent in *P*. *falciparum* cases (Table 2), in whom hepatic dysfunction was the most common complication parameter (Fig 5). Conversely, renal function parameters (creatinine and BUN levels) were normal in most cases. Although acute respiratory distress (ARDS) is also a common finding mainly in *P*. *vivax* severe cases, here we found that it was present in only 2–3% (n = 7/305) the patients, which is in agreement with recent reports from India (3%; n = 5/157), but contrast with the reports from Brazil (17.5%; n = 7/40) in the same study [12]. Whereas, in the latter study four of the patients with ARDS died, all the patients of our study successfully recovered. Additionally, all *P*. *vivax* patients were treated with Chloroquine, including the 138 who had initiated treatment at the POC, before enrolment at the hospital. In our series, Chloroquine pre-treatment appear to associate with any particular malaria complication, as previously suggested [48].

Two cases of death secondary to malaria infection were found in this study, both of them with multiple complications. Although they were recruited in Quibdó, their sites of origin were Sipi and Alto Baudó, two rural areas of Chocó that are very distant from the capital. This may represent a risk factor for the development of greater complications and death in those patients. However, the reported duration of illness was no significantly longer than in other cases.

A limitation of the present study was that qPCR confirmation of malaria parasite species was not possible in ~40% of cases, as most of the patients received treatment before their recruitment at study hospitals, and therefore qPCR was negative. Thus, recruitment of complicated malaria patients should be performed at small hospitals or local point–of-care in rural areas where patients live. However, difficulties accessing these settings make this unfeasible. Additionally, as some of the laboratory results were taken from hospital, these were not complete in all patients, particularly the biochemistry tests. This study provides valuable information for the clinical approach and monitoring of complicated malaria patients in Colombia. Based on these results, physicians in endemic settings must strategically look with special attention for haematological parameters, mainly platelet counts as severe thrombocytopenia is the main abnormal feature among complicated malaria patients regardless of parasite species. Additionally, haemoglobin levels particularly in children and P. vivax patients, and hepatic function in adults with *P*. *falciparum* infection must be primarily assessed. Future prospective studies must evaluate all the required clinical and laboratory parameters in complicated and non-complicated malaria cases in order to find clear risk factors associated with disease severity.

## Conclusions

The high prevalence of complicated malaria in the Pacific Coast of Colombia, along with more frequent severe anaemia in children infected by *P*. *vivax* and high frequency of hepatic and renal dysfunction in patients with *P*. *falciparum*, demand attention from health care professionals in order to rapidly identify and properly treat these malaria-related complications. Therefore, this study provides evidence that more conservative definitions for complicated malaria ensure a better patient management, although duration of hospitalization and ICU stay were similar in both groups criteria.

## Abbreviations

ALT: alanine aminotransferase
AST: aspartate aminotransferase
BUN: blood urea nitrogen
MoH: Minister of Health
TBS: thick blood smear
WHO: World Health Organization
